# Eight-Month-Old Infants Meta-Learn by Downweighting Irrelevant Evidence

**DOI:** 10.1162/opmi_a_00079

**Published:** 2023-06-01

**Authors:** Francesco Poli, Tommaso Ghilardi, Rogier B. Mars, Max Hinne, Sabine Hunnius

**Affiliations:** Donders Center for Cognition, Radboud University Nijmegen, Nijmegen, The Netherlands; Nuffield Department of Clinical Neurosciences, Wellcome Centre for Integrative Neuroimaging, FMRIB, University of Oxford, John Radcliffe Hospital, Headington, Oxford, UK

**Keywords:** meta-learning, infant, computational modeling, eye-tracking

## Abstract

Infants learn to navigate the complexity of the physical and social world at an outstanding pace, but how they accomplish this learning is still largely unknown. Recent advances in human and artificial intelligence research propose that a key feature to achieving quick and efficient learning is meta-learning, the ability to make use of prior experiences to learn how to learn better in the future. Here we show that 8-month-old infants successfully engage in meta-learning within very short timespans after being exposed to a new learning environment. We developed a Bayesian model that captures how infants attribute informativity to incoming events, and how this process is optimized by the meta-parameters of their hierarchical models over the task structure. We fitted the model with infants’ gaze behavior during a learning task. Our results reveal how infants actively use past experiences to generate new inductive biases that allow future learning to proceed faster.

## INTRODUCTION

Infants constantly learn from what they experience in the physical and social world around them, updating their expectations as they encounter new—and sometimes conflicting—information (Hunnius, 2022; Köster et al., 2020). These learning abilities are advanced from early in life (Emberson et al., 2015; Kidd et al., 2012; Poli et al., 2020) and possibly from birth (Bulf et al., 2011; Craighero et al., 2020). A key aspect of infant learning is the ability to exploit newly learned content to improve further learning (Dewar & Xu, 2010; Thiessen & Saffran, 2007; Werchan & Amso, 2020). However, the cognitive mechanisms that support this ability are still unknown.

This ability to learn how to learn is often referred to as meta-learning, and has been placed at the core of recent theories of human (Baram et al., 2021; Wang et al., 2018) and artificial intelligence (Lake et al., 2017). When agents meta-learn, they do not simply accumulate experiences, but actively use them to generate new inductive biases and knowledge. This in turn makes learning proceed faster and more efficiently in the future (Kemp et al., 2007; Wang et al., 2018).

A classical study by Harlow (1949) illustrates the key aspects of meta-learning. Macaque monkeys were given a choice between two objects. One of the objects predicted the location of some food with perfect accuracy, while the other object was always paired with an empty well. Every six trials the two objects (i.e., the learning set) changed, but the underlying rule remained the same: one object led to a reward, the other did not. This setup meant that in theory it was always possible to know which object was rewarded after a single trial: the chosen object if a reward was received, or the non-chosen one if no reward was received. Across hundreds of trials, the macaques learned the structure of the task and became able to predict after one single presentation of a new object set where the food was. These large timescales do not offer evidence for quick meta-learning. However, Harlow also tested preschool children with a similar task, obtaining a comparable performance in much shorter timespans (Harlow, 1949).

The study by Harlow (1949) on learning sets offers a simple but elegant example of learning to learn, a sophisticated ability that requires the agent to form meta-representations over the task space, allowing past experience with similar situations to support the learning in new situations (Wang et al., 2018). Recent advances in cognitive computational modeling capture how adults acquire structured priors that support their learning over multiple scales (Gershman & Niv, 2010; Lake et al., 2015; Poli et al., 2022; Vossel et al., 2014). Similarly, research in artificial intelligence focuses on how complex structures can emerge from statistical regularities, and what prior knowledge is required to support such processes (Alet et al., 2020; Grant et al., 2018), if any (Piantadosi, 2021). Such questions are extremely relevant within developmental science (Rule et al., 2020; Xu, 2019; Yuan et al., 2020), and Bayesian theories of brain functioning (Tenenbaum et al., 2011) hold promise to answer them in terms of hierarchical priors (or overhypotehses) that can be either learned or refined with the accumulation of new knowledge. This would help overcome the rigid dichotomy of “empiricism versus nativism”, moving towards a better understanding of the nature of the cognitive mechanisms that underlie learning in infancy and of the priors that might support acquisition of knowledge from birth.

From the early studies on learning sets (Harlow, 1949; Koch & Meyer, 1959), developmental science has come a long way. Infants’ learning skills (Emberson et al., 2015; Romberg & Saffran, 2013; Trainor, 2012), their flexibility (Kayhan et al., 2019; Tummeltshammer & Kirkham, 2013) and complexity (Werchan et al., 2015) have been widely demonstrated. However, current research lacks a mechanistic explanation of how meta-learning occurs in the infant mind. The key difference between meta-learning and other forms of learning is that meta-learning does not change the infants’ models of the world directly, but the very same learning processes that allow them to further shape their internal models of the world. In this paper, we show how we can leverage hierarchical Bayesian models to gain novel insights into the cognitive mechanism that allows infants to exploit prior knowledge to optimize how new stimuli are learned. We studied 8-month-old infants because at this age infants have already started to develop the ability to control their attention and to actively disengage from a stimulus, a behavioral measure that plays an important role in our analyses.

We presented infants with multiple probabilistic sequences of stimuli while monitoring their eye movements via eye-tracking, and we quantified the information gain of each stimulus using Bayesian updating and information theory. In our task, information gain refers to the degree to which every stimulus contributes to effectively learning the probabilistic structure of the task. We propose that when infants meta-learn, they gain the ability to strategically weight the value of incoming information. Namely, they might upweight the information content of stimuli that are expected to carry more information, or downweight the information content of stimuli that are expected to be irrelevant. Similar to Harlow’s (1949) task, our task provided a general structure that infants could extract over multiple sequences. In each sequence, the most relevant information was in the first trials, and later trials were less relevant to learn to predict the following stimuli. If infants understood this structure, they could make use of it to change how they learned: By upweighting evidence acquired early in the sequence and downweighting evidence acquired later on, they could optimize their learning. We expected this to be reflected in increased looking times to the initial stimuli that offer more information, and increased probability of disengaging from the screen for the stimuli that are less relevant for learning.

## METHODS

### Participants

Ninety 8-month-old infants (*M* = 8.05 months, *SD* = 11.56 days, 42 females) were recruited for this study from a database of volunteer families. Infants that carried out less than 20 trials were excluded from the analysis (*N* = 17) as they did not perform enough trials in at least two sequences. The final sample consisted of 73 infants (*M* = 8.02 months, *SD* = 11.37 days, 34 females). For this study, a previously published dataset (Poli et al., 2020) was reanalysed (*N* = 50), and new data were collected using the same task (*N* = 40). Since the previous study focused on a different set of hypotheses, familiarity with the existing dataset did not impact hypothesis formation.

### Procedure

Infants were tested in a silent room with fixed levels of light. They were placed in a baby seat, which was held by the parents on their lap. The eyes of the infants were approximately 65 cm far from the screen. Parents were instructed not to interact with their child, unless infants sought their attention and, even in that case, not to try to bring infants’ attention back to the screen. During the experiment, the infants’ looking behavior was monitored using both a Tobii X300 eye-tracker and a video camera. We extracted visual fixations using I2MC (Hessels et al., 2017) with the default parameters of the algorithm. We then extracted saccadic latency, looking time and look-away from the pre-processed data.

### Experimental Paradigm

**Figure 1.**
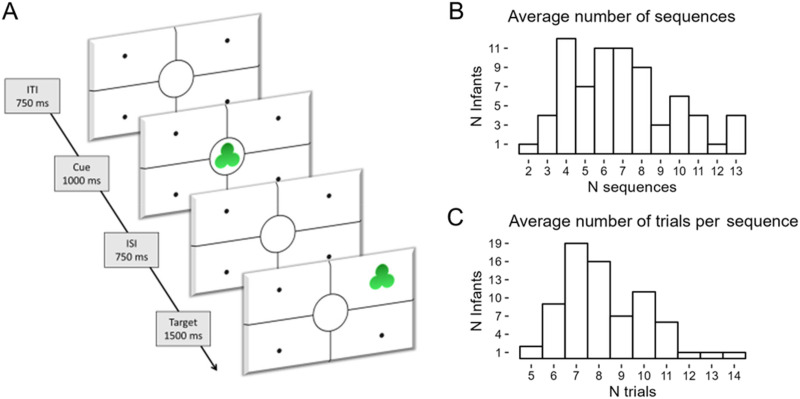
**(A) An example trial of the visual learning task.** Each sequence was composed of a maximum of 15 trials and terminated when infants looked away for 1 second. ITI = Inter-Trial Interval, ISI = Inter-Stimulus Interval. (B) Descriptive statistics of the average number of sequences watched by each infant. (C) Descriptive statistics of the average number of trials watched in each sequence by each infant.

**Figure 2.**
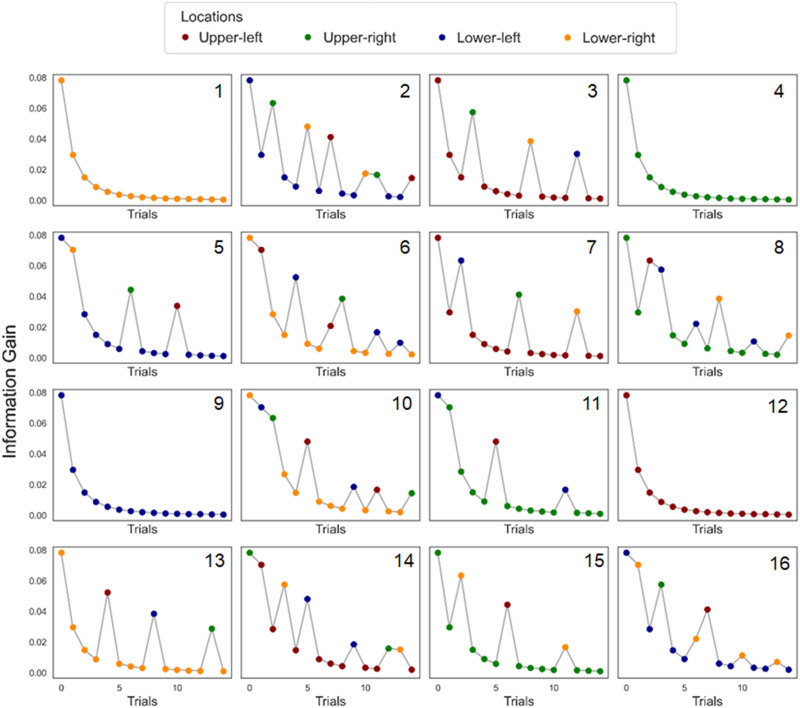
**Estimates of information gain for all the sequences of the visual learning task.** These estimates were used as input in the model, and the data from infants (looking times, saccadic latencies, and look-aways from the screen) informed the model about whether information was up- or downweighted across time. Colours indicate the four target locations (pseudo-randomized across participants). The number of each sequence is given in the upper-right corner of every plot. The location of the stimuli was pseudo-randomized, so that each location has the same overall probability of showing the target (25%), and each probabilistic sequence contains more than one low-probability location.

Infants were presented with a visual learning eye-tracking task composed of 16 sequences of stimuli. Every sequence contained 15 cue-target pairs (i.e., trials), where the cue was a shape appearing in the middle of the screen, and the target was the same shape appearing in one of four screen quadrants around the cue location (Figure 1). The shape was the same across all trials of the same sequence but changed across sequences. For all sequences, the target could appear in any location, but it appeared in one specific location more often than the others. Specifically, it appeared in the high-likelihood location 60% of the times in 6 sequences, 80% of the times in 6 other sequences, and 100% of the times in 4 sequences. A representation of all sequences in their order of presentation is given in Figure 2. The sequences were shown one after the other in the same order for all participants. When the infant looked away from the screen for one second or more, the sequence was stopped. When the infant looked back to the screen, the following sequence was played. The experiment lasted until the infant had watched all 16 sequences or became fussy. This procedure may result in non-random missingness of the data (e.g., fewer datapoints for later sequences). Additional information can be found in the Supplementary Materials.

### Computational Model

We developed a cognitive Bayesian model of infants’ looking behavior that exploits the richness of eye-tracking data to infer the values of latent parameters capturing how infants processed the information content of the stimuli over time. To do so, we engaged in a ‘reverse-engineering’ procedure: given the data, we wanted to infer information about infants’ cognitive processes (i.e., the latent parameters of the model). A consequence of this approach is that we were not constrained to the use of only one dependent variable, as is often the case in statistical models. Instead, we exploited multiple dependent variables to improve the model estimates of the latent parameters (e.g., Lee & Wagenmakers, 2014). Specifically, we collected three variables from the infants’ looking behavior that have been shown to relate to information processing (Kidd et al., 2012; O’Reilly et al., 2013; Poli et al., 2020): 1) *Look-Away*. For each trial, we recorded whether infants kept looking at the screen or looked away; 2) *Saccadic Latency*. We measured how quickly infants moved their eyes from the cue to the target location, from the moment the target appeared. Negative times (i.e., anticipations to the target location) were also possible; 3) *Looking Time*. We measured how long infants looked at the target location, from the moment it appeared to 750 ms after its disappearance.

#### Bayes-optimal learning.

In every trial *t* of a sequence *s*, a stimulus is shown in the target location *x*_*s*,*t*_ ∈ {1, …, *K*}, where *K* = 4. Starting from the initial uniform prior *γ*_*s*_ = [1, 1, 1, 1], which assumes that the target is equally likely to appear in any of the four locations (i.e., 25%), the probability *P*(*X*)_*s*,*t*_ of seeing the stimulus in any given location is updated in light of the new evidence *x*_*s*,*t*_:

$$P{\left(X\right)}_{s,t}=\frac{X_{s,t}+\gamma_{s}}{t+\text{K}}$$

where *X*_*s*,*t*_ indicates all the evidence that has been observed up until trial *t*. From these probabilities, we quantified how much information the stimulus was conveying using the Kullback-Leibler (KL) Divergence (Kullback & Leibler, 1951), or *D*_*KL*_:

$$D_{KL}=\sum_{\text{i}=1}^{\text{K}}P{\left(X\right)}_{s,t}\log\left(\frac{P{\left(X\right)}_{s,t}}{P{\left(X\right)}_{s,t-1}}\right)$$

KL-Divergence captures how much the new stimulus changed the probability distribution of the events or, in other words, how much information is gained from the new stimulus to understand the underlying (hidden) probability distribution of the events. We chose this approach to quantifying information gain for its simplicity and for its wide use within the field of cognitive neuroscience (Mars et al., 2008; O’Reilly et al., 2013). The resulting estimates are reported in Figure 2.

**Figure 3.**
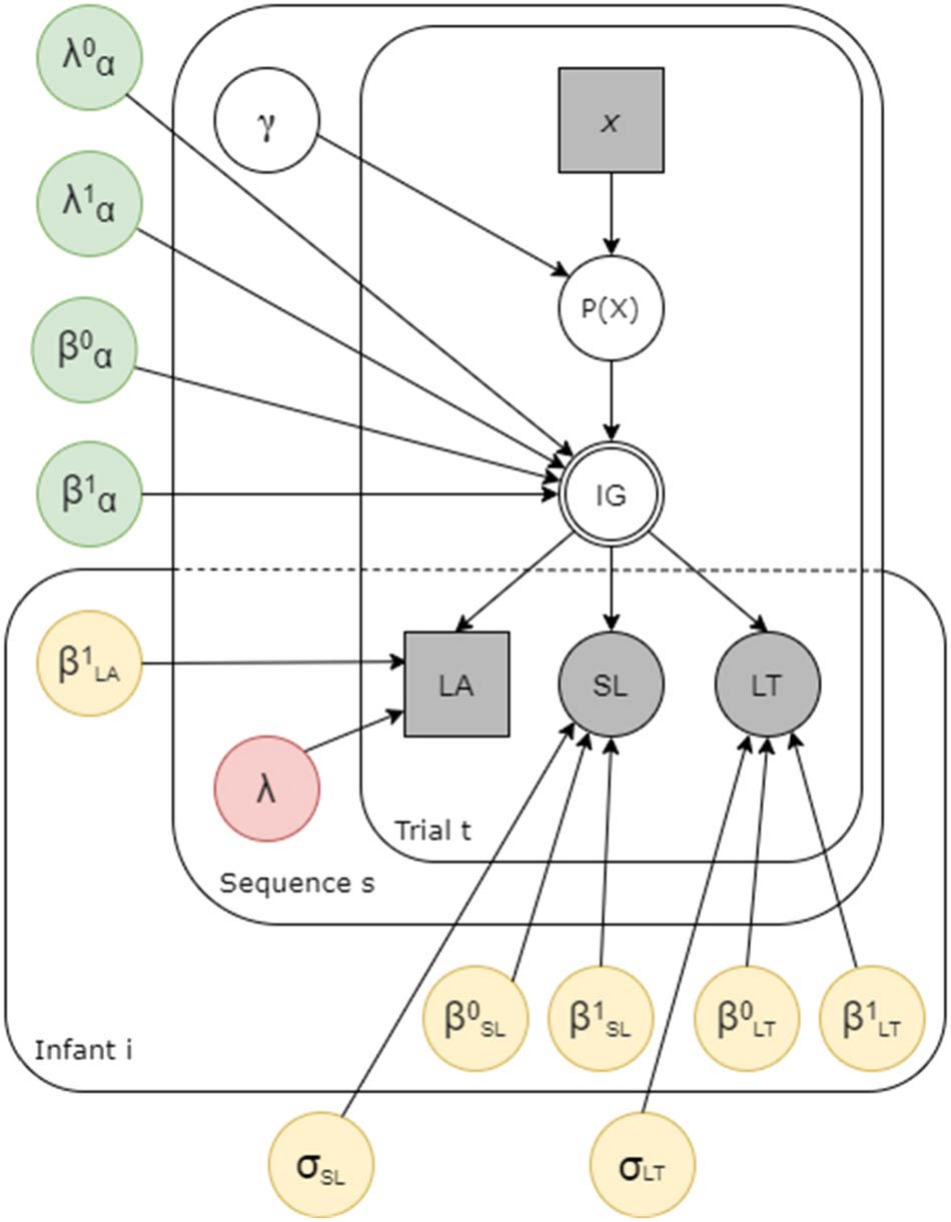
**The generative model.** Squares indicate discrete variables and circles indicate continuous variables; Grey boxes indicate observed variables; In green, parameters regulating the up- and downweighting of information; In yellow, parameters accounting for individual differences across infants; In red, the parameter controlling for infants’ changing attention across sequences. Note that hyperparameters and parameters controlling covariates are not reported in this figure, but are available in the Supplementary Materials.

To test our hypotheses on up- and downweighting of information, we introduced an exponential decay of information gain over trials (see Figure 3, in green). The decay could vary across sequences, thus allowing information on early sequences to be processed differently from information acquired later in the task. The decay was regulated by four additional parameters $\lambda_{\alpha }^{0}$, $\lambda_{\alpha }^{1}$, $\beta_{\alpha }^{0}$, and $\beta_{\alpha }^{1}$, such that:

$${IG}_{s,t}=\underset{\text{Early}\;up-\text{weight}}{\underbrace{\left(\lambda_{\alpha }^{0}+\lambda_{\alpha }^{1}s\right)}}\;\underset{\text{Late}\;\text{down}-\text{weight}}{\underbrace{\exp(-t\left(\beta_{\alpha }^{0}+\beta_{\alpha }^{1}s\right)}}\;D_{{KL}_{s,t}}$$

where $\lambda_{\alpha }^{0}$ and $\lambda_{\alpha }^{1}$ regulate the upweighting across sequences of the information acquired in trials early in the sequence, while $\beta_{\alpha }^{0}$ and $\beta_{\alpha }^{1}$ regulate the downweighting across sequences of the information acquired in trials late in the sequence. The parameters $\lambda_{\alpha }^{0}$ and $\beta_{\alpha }^{0}$ are simple intercepts, while $\lambda_{\alpha }^{1}$ and $\beta_{\alpha }^{1}$ are the regression coefficients of interest, and our focus will thus be on the latter. If meta-learning occurs, we would expect that as sequences are observed, infants start to extract the underlying informational structure. As a consequence, the more sequences they are exposed to, the more they would upweight the information acquired in the first trials or downweight the information of late trials of each sequence. These two hypotheses are not mutually exclusive: Infants might both upweight early evidence and downweight late evidence. This would translate in the model returning positive values for both $\lambda_{\alpha }^{1}$ and $\beta_{\alpha }^{1}$. Finally, we introduced parameters to control the effect of time on infants’ behavior. First, the parameter *λ*_*s*_ controls for changes in baseline attention to the task across sequences (see Figure 3, in red). This allows us to disentangle whether changes in attention across sequences are in fact due to meta-learning or simply related to fatigue. Second, when estimating the relation of information gain with the dependent variables (saccadic latency, looking time, and look-away) we always included the trial number as covariate (see Supplementary Materials). Hence, any correlation we find between information gain and these variables was obtained while accounting for changes in behavior over time.

We modeled how the information content in the stimuli was related linearly to the different observed variables, Saccadic Latency (SL), Looking Time (LT), and Look-Away (LA), in a probabilistic way. When estimating the relationship between information gain and the dependent variables, the regression coefficients (see Figure 3, in yellow) are estimated hierarchically, both at the group level and for each participant, thus taking into account between-subject variability. Further specifications of the priors and likelihoods of the model are available in the online Supplementary Materials.

#### Inference.

The model was fitted in Python using PyMC3 (Salvatier et al., 2016). Two chains of 400,000 samples each were run using No-U-Turn-Sampler (NUTS), a common gradient-based MCMC algorithm. 390,000 samples were discarded as burn-in, and the last 10,000 samples were kept for further analysis. All the parameters reached convergence, as indicated by $\hat{R}$ < 1.05 for all parameters (see Gelman & Rubin, 1992).

## RESULTS

### Model Comparison

We compared the full model to reduced models that did not include some (or all) of the parameters modulating the up and down-regulation of information. The upweight model assumed that, as sequences accumulated, information early in the sequences was progressively upweighted. The downweight model assumed that, as sequences accumulated, information late in the sequences was progressively downweighted. The full model assumed concurrent up- and downweighting of information, while the no-weight model assumed no change across sequences in the way information gain decayed over trials. The negative expected log predictive densities (ELPD) of the models were compared with Leave-One-Out (LOO) and WAIC methods.

The results of model comparison are reported in Table 1. As expected, LOO and WAIC methods returned similar estimates for all of the dependent variables. When comparing the total scores of the different models, the downweight model and the full model performed better than the null model and the upweight model, as indexed by lower negative ELPD scores (Kruschke, 2011). The difference in performance between the downweight model and the full model was negligible. The upweight parameter did not affect the results in either of the two models: it was absent in the downweight model, and it was not different from zero for the full model (see below and in Figure 5). Hence, both models support the conclusion that downweighting is present, while upweighting is not.

**Table 1.** Model comparison between the full model and different reduced models, for each dependent variable and as a whole.

Model	Dependent Variable	-ELPD LOO	ΔLOO	-ELPD WAIC	ΔWAIC
Null model	Looking Time	4686		4685	
	Saccadic Latency	4555		4555	
	Look-Away	1201		1201	
	**Total**	**10442**	**0**	**10441**	**0**
Upweight	Looking Time	4683		4683	
	Saccadic Latency	4552		4552	
	Look-Away	1205		1205	
	**Total**	**10440**	**2**	**10440**	**1**
Downweight	Looking Time	4675		4674*	
	Saccadic Latency	4549		4549*	
	Look-Away	1194		1194	
	**Total**	**10418**	**24**	10418*	**23**
Full model	Looking Time	4675		4675	
	Saccadic Latency	4549		4549	
	Look-Away	1192		1192	
	**Total**	10416*	26*	10416*	25*

*Note*. Delta values indicate the difference from the null model. Asterisks indicate the best models.

### Information Gain Predicted Infants’ Looking Behavior

Before analysing the parameters of interest (i.e., the up- and downweight parameters), we examined whether the dependent variables were significantly related to the information gain estimates. This step is crucial as we assumed that the dependent variables can inform latent parameters related to how information is processed. We found that the beta coefficients relating the dependent variables to information gain were different from zero for look-aways (mean = −4.98, *SD* = 0.61, 94% HDI = [−6.21, −3.87]), looking time (mean = −0.57, *SD* = 0.20, 94% HDI = [−0.99, −0.20]) and saccadic latency (mean = 0.69, *SD* = 0.19, 94% HDI = [0.34, 1.05]), and none of the credible intervals included zero. Hence, the ‘reverse-engineering’ procedure was successful, as the dependent variables were all contributing to inform the levels of information content and their change over time.

**Figure 4.**
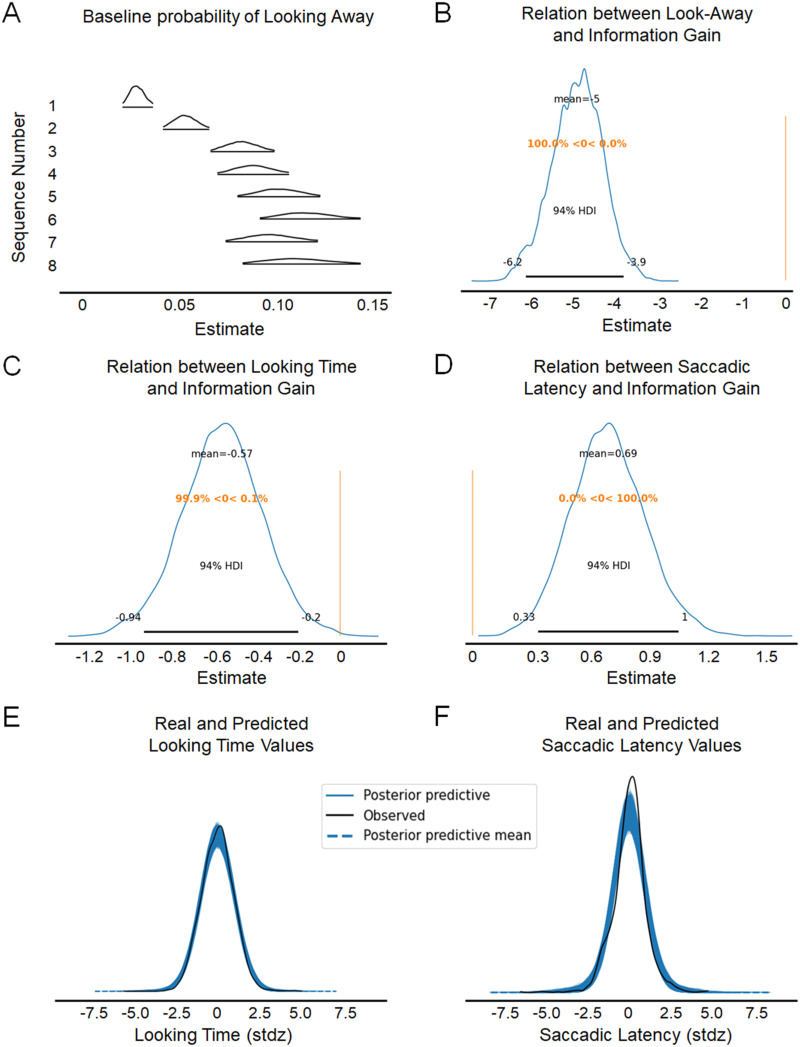
**Results for the coefficients relating information gain to looking behavior.** (A–D) The peak of the distributions indicate the most likely value for the parameter, and the rest of the curve indicates other possible values and their relative likelihood. (E–F) The posterior predictive distributions for looking time and saccadic latency consists of multiple overlapping lines obtained by generating the data multiple times after the model was fit.

To check whether our model was a good fit to the data, we performed a posterior predictive check. In other words, the values of the posterior distributions were used to predict new data, thus testing whether they could produce reliable estimates. The real data and the predicted distribution of the data matched well (Figure 4).

### Infants Meta-Learn by Downweighting Irrelevant Information

**Figure 5.**
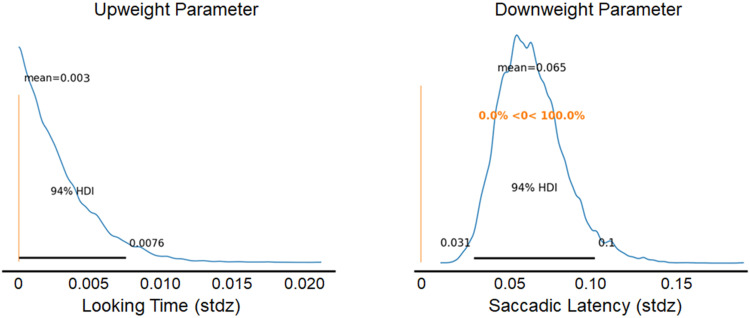
**The posterior distributions for $\lambda_{\alpha_{s}}^{1}$ and $\beta_{\alpha }^{1}$**. The peaks of the distributions indicate the most likely values for the parameters, and the rest of the curve indicates other possible values and their relative probability. The parameters $\lambda_{\alpha_{s}}^{1}$ and $\beta_{\alpha }^{1}$ regulate the up- and downweighting of information gain, respectively.

Table values of the parameters $\lambda_{\alpha }^{1}$ and $\beta_{\alpha }^{1}$ accounted for the up- and down-regulation of information across sequences. The upweight parameter ($\lambda_{\alpha }^{1}$) was not different from zero (mean = 0.003, *SD* = 0.003, 94% HDI = [0, 0.009]), confirming that there was not a significant relationship between upweighting of information found early in a sequence and sequence number. The parameter $\beta_{\alpha }^{1}$ was different from zero (mean = 0.065, *SD* = 0.019, 94% HDI = [0.031, 0.102]), confirming that there was a significant relationship between downweighting of information found late in a sequence and sequence number (Figure 5).

**Figure 6.**
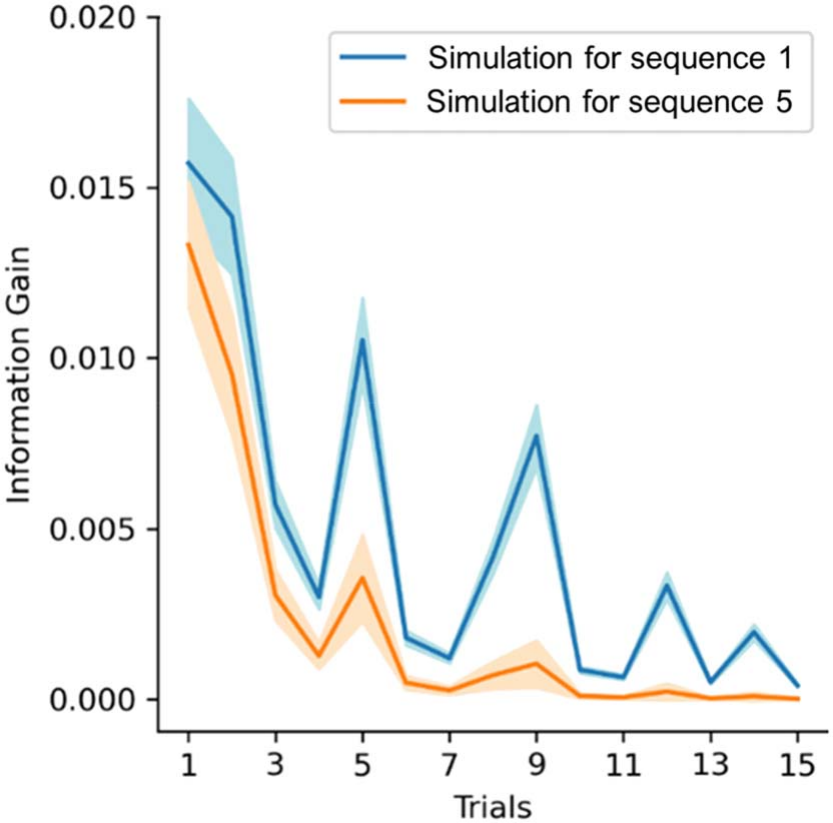
**Simulation of information gain values for an early and a late sequence.** After the model was fit to the data, the resulting parameters estimates were used on a novel unobserved sequence (Sequence 16) to simulate information gain values at different time points (i.e., for the 1st and 5th sequence). On average, infants watched 7 sequences. Compared to the blue line, the orange line is not higher at the start of the sequence (information is not upweighted) but it is lower in following trials (information is downweighted). Shaded areas indicate 95% credible intervals.

To better understand how the parameters capture infants’ attribution of informativity to incoming stimuli, we simulated the up- and downweighting of information gain on a novel unseen sequence (i.e., sequence 16, see Figure 2). We used the mean values of the parameters estimated by the model that was fit with infants’ gaze data (as reported in Figure 4 and 5) to generate the perceived values of information gain if this had been presented early (as sequence 1) or late (as sequence 5) during the experiment. The results are reported in Figure 6. Comparing an early sequence to a late sequence, the information gain of the first trials did not change as the sequences progressed, while information gain of trials later on in the sequence was downweighted for late sequences. This effect was present while controlling for changes in baseline attention across sequences (see *λ*_*s*_ in Figure 4).

### Infants’ Learning Efficiency Improves Over Time

Once we identified the mechanisms underlying infants’ meta-learning, we examined whether infants achieved more efficient and faster learning over time. To test this, we analysed saccadic latencies for predictable trials. The first trial of each sequence was excluded and only trials that showed the most predictable location are analysed. When a target repeatedly appears in the same predictable location, saccadic latencies to the target should decrease. Moreover, in later sequences, if infants are exploiting what they meta-learned from previous sequences, saccadic latencies should be even shorter from earlier on. We analysed saccadic latencies as a linear function of sequence number and as a logarithmic function of trial number, while controlling for overall trial number. Participants were included as random intercepts, and we included random slopes for all predictors that vary within participants (sequence number, log within-sequence trial number, their interaction, and overall trial number). The model was run on R with brms (Bürkner, 2017) with two chains of 10,000 samples, and the first 5000 samples were discarded as burn-in.

**Figure 7.**
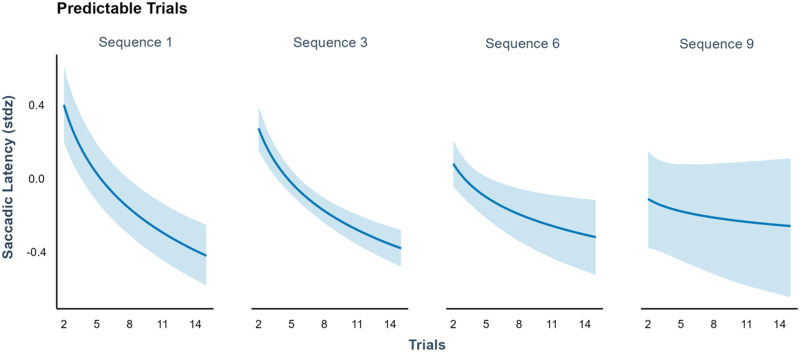
**Saccadic latencies as a function of sequence and trial number.** Saccadic latencies for predictable trials decreased across time, indicating a learning effect. This effect changed across sequences, as later sequences presented faster saccadic latencies from early on, suggesting that meta-learning led to more efficient learning. Shaded areas indicate 95% credible intervals.

We found a main effect of trial number (*β* = −0.45, *SD* = 0.05, 94% HDI = [−0.56, −0.33]), indicating that infants were learning the predictable target locations successfully. More importantly, we found an interaction between sequence number and trial number (*β* = 0.04, *SD* = 0.01, 94% HDI = [0.01, 0.07]) due to differences across sequences in saccadic latency to early trials (as indicated by significant marginal effects for trial 2, *β* = −0.06, 94% HDI = [−0.12, −0.01]). This indicates significantly lower values of saccadic latencies for early trials of late sequences, which supports the idea that learning was faster for later sequences, just as predicted by our meta-learning results (Figure 7).

## DISCUSSION

Meta-learning is a complex ability that entails building and tuning meta-parameters over a task to shape future expectations. Whereas it was known that infants’ learning is incremental and that infants build new knowledge relying on what they have learned in the past (Thiessen & Saffran, 2007), the cognitive mechanisms underlying meta-learning were still unknown. In the current study, we show that infants can meta-learn from limited evidence on a relatively small timescale (i.e., within minutes). More importantly, computational modeling demonstrates *how* they achieve this meta-learning: Infants identify where in the environment information is, and they use this knowledge to downweight the informativity of irrelevant stimuli. Conversely, we do not find evidence supporting the idea that they upweight the informativity of the relevant stimuli. In addition, we find that as time progressed, infants needed less information and their learning efficiency increased, as indexed by faster saccadic latencies.

These results were obtained while controlling for the effect of fatigue and for the passage of time, and thus cannot be explained by a simple decrease in interest in the stimuli over time. However, future studies should be designed decreasing as much as possible the non-random nature of missing data. Moreover, future research should explore whether the current findings can be generalized across paradigms and contexts. In the current paradigm, the informative evidence was always at the start of the sequence, and the very first sequence was always 100% predictable. Although this does not invalidate the evidence for downweighting of information as we find it, different structures (e.g., with information that is delivered mid-way through the sequence) as well as different orders of sequences should be examined in the future to determine the generalizability of our results. More broadly, it is not known how infants apply these learning strategies in real life learning.

Although we find that infants meta-learn by downweighting irrelevant evidence, we do not find an upweighting of the relevant evidence. One possibility is that at 8 months of age, infants are still unable to perform this upweighting process, which has yet to emerge later in life. This idea is consistent with the possibility that meta-learning mechanisms might not be in place from birth and develop across longer timescales instead (Wang et al., 2018). Testing multiple age ranges in the future will enhance our understanding of the exact development of meta-learning skills. Another possibility is that upweighting as a part of meta-learning is never instantiated in the human mind, neither in infants nor in adults. In fact, whereas we did not find evidence that relevant information was upweighted and thus gained importance in absolute terms, downweighting of irrelevant information still enhances that information in relative terms. This may be enough for cognitive systems to orient their attention and information processing resources efficiently. Future research should investigate meta-learning in adults with comparable computational models to clarify this issue.

The idea that infants learn inductive biases that change their information processing over time is in line with the predictive processing framework. Predictive processing holds that the brain is a hierarchically-structured prediction engine, with prediction travelling from higher to lower levels in the hierarchy, and prediction errors moving in the opposite direction, from lower to higher levels (Clark, 2013). In predictive processing terms, prediction errors are precision weighted, that is, their importance is modulated depending on their relevance. The down-weighting of information content as we find it is assimilable to the predictive processing concept of precision-weighting of the prediction error. Specifically, in later sequences, the structure of the task is already clear to the infants, so new information is less likely to change their expectations, and as a consequence, it is downweighted. Thus, the current study contributes to recent literature on infant development suggesting that the foundations of a predictive mind are present from early on in life (Köster et al., 2020).

The ability to meta-learn is crucial for learning optimization and the acquisition of new abilities (Yoon et al., 2018). Its presence in the first year of life is in line with the idea that complex functions can emerge dynamically from the interaction between powerful learning mechanisms (Chen & Westermann, 2018; Poli et al., 2020; Silverstein et al., 2021), fundamental attentional biases (Di Giorgio et al., 2017), and early accumulation of evidence (Craighero et al., 2020; Ossmy & Adolph, 2020). Recent theoretical and empirical work favours the idea that high-level cognitive systems can be generated via sub-symbolic dynamics (Piantadosi, 2021; Sheya & Smith, 2019). For example, Yuan et al. (2020) investigated how preschoolers learn to map multi-digit number names onto their written forms. They presented children and a deep learning neural network with minimal training material and showed that both children and machines could reach systematic generalizations from limited evidence. Hence, complex and symbolic structures can emerge from simple mechanisms, and meta-learning might greatly ease this process from a very young age. The key idea is that some brain networks might work on long timescales to train other networks that are faster and more flexible. For example, the sub-cortical dopaminergic system might train prefrontal areas to function as free-standing learning system (Wang et al., 2018). This hypothesis has only recently gained interest in the adult literature (Dehaene et al., 2022), and its predictions about the developmental pathways of (sub)symbolic representations remain to be tested.

Finally, the current work offers a new methodological approach to infant research. Usually, statistical models try to link one or more independent variables to a single dependent variable by fitting a number of parameters (Lee, 2018). Conversely, in the computational model that we designed, the values of latent parameters were informed by multiple sources of data. In our case, saccadic latency, looking time to the targets, and looking away from the screen contributed to informing the relevant latent parameters. This approach is especially useful in infant research, which is often characterized by smaller datasets with lower signal-to-noise ratios compared to adult research (Havron et al., 2020). The limitations inherent to infant research can thus be compensated using richer measurements and dedicated computational models.

## ACKNOWLEDGMENTS

We thank the lab managers and the student assistants for helping with data collection, and all the families that participated in our research.

## AUTHOR CONTRIBUTIONS

F.P.: Conceptualization, formal analysis, investigation, methodology, and writing – original draft. T.G.: Conceptualization, formal analysis, writing – review and editing. R.B.M: Methodology, supervision, writing – review and editing. M.H.: Formal analysis, supervision, writing – review and editing S.H.: Methodology, supervision, writing – review and editing.

## FUNDING INFORMATION

This work was supported by the Donders Centre for Cognition internal grant to S.H. and R.B.M. (“Here’s looking at you, kid.” A model-based approach to interindividual differences in infants’ looking behaviour and their relationship with cognitive performance and IQ; award/start date: 15 March 2018), BBSRC David Phillips Fellowship to R.B.M. (“The comparative connectome”; award/start date: 1 September 2016; serial number: BB/N019814/1), Netherland Organization for Scientific Research NWO to R.B.M. (“Levels of social inference: Investigating the origin of human uniqueness”; award/start date: 1 January 2015; serial number: 452-13-015), Wellcome Trust center grant to R.B.M. (“Wellcome Centre for Integrative Neuroimaging”; award/start date: 30 October 2016; serial number: 203139/Z/16/Z), and Dutch Research Council NWO to S.H. (“Loving to learn - How curiosity drives cognitive development in young children”; start date: 1 February 2021; serial number: VI.C.191.022).

## DATA AVAILABILITY STATEMENT

Experimental data and computational models can be found on OSF: https://osf.io/a93qr/.
