# Supplementary material for: Eight-Month-Old Infants Meta-Learn by Downweighting Irrelevant Evidence
Source: Open Mind (Camb). 2023 Jun 1;7:141–55. doi: 10.1162/opmi_a_00079 (PMC10320826; doi:10.1162/opmi_a_00079)
Supplement: Supplementary file 1 [file opmi-07-141-s001.pdf]

Supplementary Materials

Raw data. Figure 3 shows the raw data for Looking Time and Saccadic Latency, divided based on predictable and unpredictable trials. Trials were classified as predictable if the target was presented in the high-likelihood location, and unpredictable if they were presented somewhere else. We found a main effect of predictable vs. unpredictable trials (while controlling for overall time, sequence number and trial number) for saccadic latency (β = -.19, 94%HDI = [-0.27, -0.12]) and look-aways (β = 0.37, 94%HDI = [0.10, 0.66]), but not looking times (β = 0.001, 94%HDI = [-0.04, 0.05]). This supports the

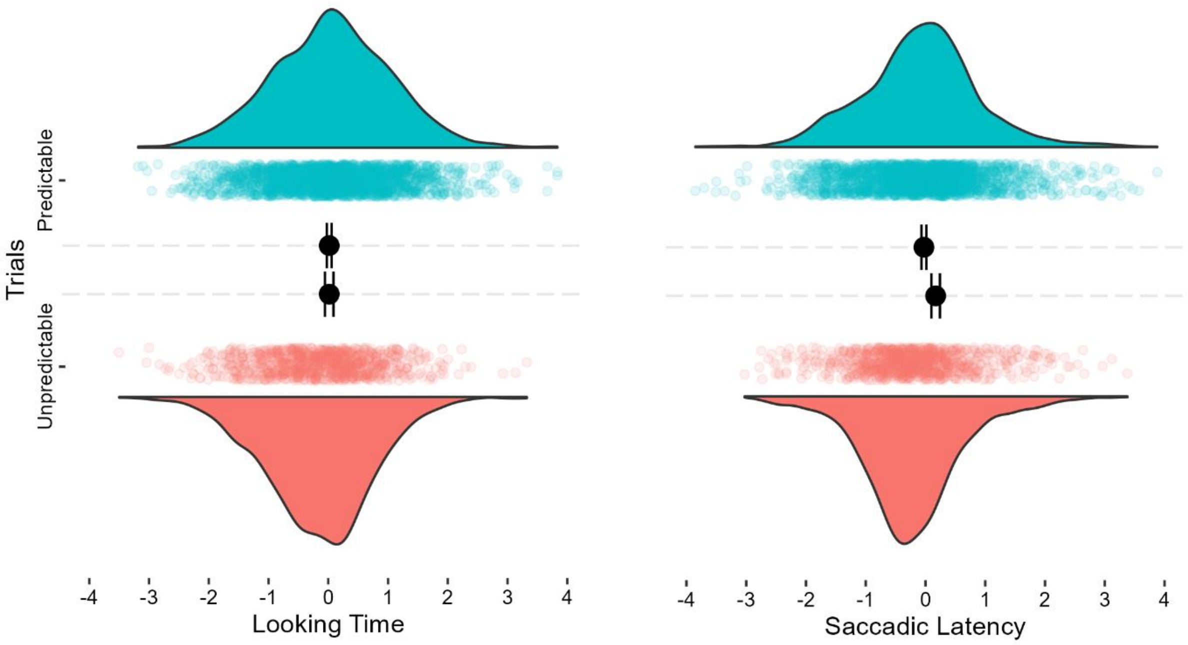

idea that these data can inform the latent parameters of our computational model. However, our model captures predictability and information gain in a more fine-grained fashion, as introduced in the main paper.

Supplementary figure 1. Distribution of standardized looking time and saccadic latency divided between predictable and unpredictable trials, with jittered datapoints and predictive means (in the centre)

Before proceeding with the modelling work, we assessed data missingness. Given the structure of our task, as time progresses, more data is missing (i.e., infants disengage earlier in later sequences. Although low and non-random data missingness should always be preferred, this is seldom the case in infant studies (Byers-Heinlein et al., 2022), especially when participants are always free to decide whether to allocate their attention. Two problems that might arise from non-randomness in the missingness of the data are that: 1) there are not enough trials to achieve reliable estimates for later sequences and 2) infants are ”self-selecting” out of the dataset as the experiment unfolds. This, in turn, might result in saccadic latency being faster in later sequences *because* only infants with fast latencies make it to the end of the task.

We addressed the first problem by checking the extent to which infants watched fewer trials for later sequences (Poisson generalized linear model). We found that, although data for later sequences were fewer (β = -0.04, 94%HDI = [-0.06, -0.03]), they still showed a considerable number of trials (see supplementary figure 2 for expected means), which should allow for good estimates also for later sequences. To adjust for these changes across trials and sequences, we will consider trial and sequence number when estimating the effects of interest.

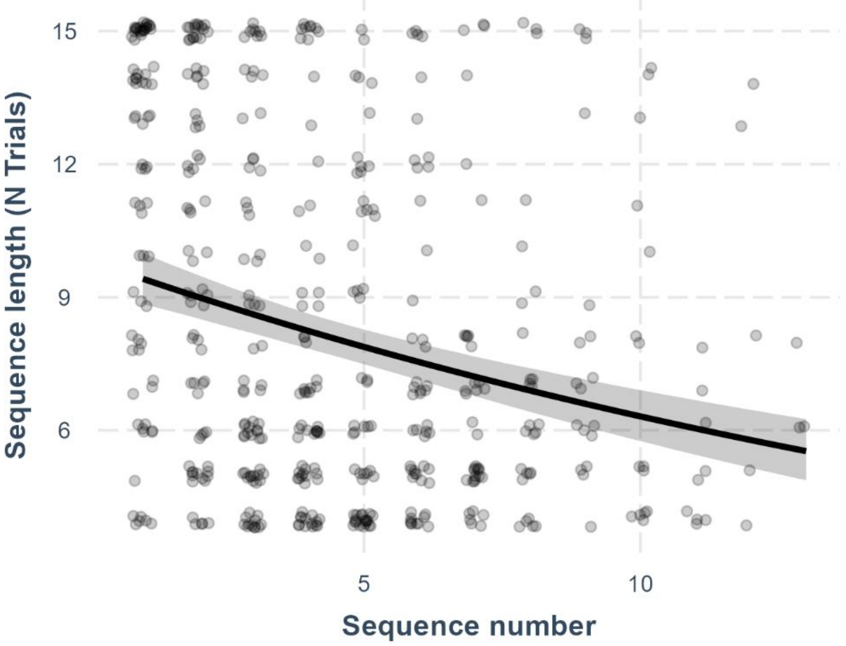

Supplementary Figure 2. As sequences progress (x-axis), infants watch less trials (y-axis).

We addressed the second problem by checking whether infants with faster saccadic latencies or shorter looking times watched a higher number of trials (Poisson generalized linear models). We do not find evidence for a relationship between sequence number and either individuals' average saccadic latency (β = -0.00006, 94%HDI = [0, 0]) or looking time (β = -0.0001, SD =, 94%HDI = [0, 0]).

In summary, the current assessment of data randomness, together with the control of time-varying covariates and the model comparison procedures described below, indicate that the model results cannot be ascribed to the non-random missingness of data alone.

Model priors. We report the prior distributions of all the parameters in the model. It must be noted that we always preferred vague priors (i.e., high variance), allowing the model to be predominantly informed by the data. The parameters λ_α^0, λ_α^1, β_α^0, and β_α^1 are assumed to follow a half-Normal distribution, which constrains them to take only values equal or greater than zero:

$$\lambda_\alpha^{\mathrm{j}} \sim \mathrm{HalfNormal}(0,1) \qquad j \in \{0,1\}$$

$$\beta_\alpha^{\mathrm{j}} \sim \mathrm{HalfNormal}(0,1) \qquad j \in \{0,1\}$$

This is a constraint due to the fact that KL-Divergence is a non-negative score, and thus it must scale with nonnegative terms. The half-Normal distribution was chosen to ease the interpretation of these parameters as regression coefficients. Similarly, hazard (which is proportional to a probability) cannot be negative, and thus the parameter λ_s has a Gamma distribution, which can only take positive values:

$$\lambda_s \sim \text{Gamma}(\kappa, \theta)$$

Where values of 0 would indicate that infants never look away from the screen, and the likelihood of looking away increases as λ values increase. A different value of λ was estimated for each sequence s, and the general values across sequences were captured by the hyperpriors κ and θ:

$$\kappa \sim \text{Gamma}(1,1)$$

$$\theta \sim \text{Gamma}(1,1)$$

We chose Gamma distributions following Ibrahim, Chen, and Sinha (2001). This prior requires the partitioning of the time range in question into intervals, and it thus fits well with the trial-by-trial structure of our design. All β_* for the regressions follow normal distributions, and they are thus free to detect both positive and negative relationships between the independent variable (i.e., information gain) and the data:

$$\beta_{LA_i} \sim \text{Normal}(\mu_{LA}, 1)$$

$$\beta^{j}_{SL_i} \sim \text{Normal}(\mu_{SL}, 1) \qquad j \in \{0,1\}$$

$$\beta^{j}_{LT_i} \sim \text{Normal}(\mu_{LT}, 1) \qquad j \in \{0,1\}$$

The values of β_* were estimated hierarchically. The mean estimate for each participant was inferred starting from the hyperparameters μ_*, that also followed a normal distribution:

$$\mu_* \sim \text{Normal}(0,1)$$

Model likelihood. The priors were linked to the data (i.e., the likelihoods) using a Student-t distribution for Saccadic latency (SL), and a normal distribution for Looking Time (LT); Look-Away (LA) is modelled with a piecewise-constant proportional hazard model with a Poisson likelihood (Rodríguez, 2008):

$$LA_{i,s,t} \sim \text{Poisson}(\lambda_s \exp(\beta_{LA_i} IG_{\text{i,s,t}}))$$

$$SL_{i,s,t} \sim \text{Norm}(\beta^0_{SL_i} + \beta^1_{SL_i} IG_{\text{i,s,t}} + \beta^2_{SL_i} t,\ \sigma_{SL})$$

$$LT_{i,s,t} \sim \text{T} - \text{Student}(\beta^0_{LT_i} + \beta^1_{LT_i} IG_{\text{i,s,t}} + \beta^2_{LT_i} t,\ \sigma_{LT}, \text{v} = 15)$$

Saccadic latency and looking time were standardized across participants and at the group level. The standard deviation terms σ_{SL} and σ_{LT} have half-Cauchy distributions, which again constrains their values to be nonnegative, as standard deviations cannot take negative values:

$$\sigma_{SL} \sim \text{halfCauchy}(0,1)$$
$$\sigma_{LT} \sim \text{halfCauchy}(0,1)$$

We chose half-Cauchy distributions because they are recommended by Gelman (2006) for modelling standard deviations, given the advantage of being approximately uniform in the tail and weak near zero.

Model comparison. Here, we assess additional models that favour alternative explanations of the results, and/or control for potential issues with the multivariate data. First, we checked whether the effect of meta-learning could be observed over and above the effect of change in saccadic latency and looking time across sequences. We added a covariate to both regressions, thus obtaining the following:

$$SL_{i,s,t} \sim \text{Norm}(\beta^0_{SL_i} + \beta^1_{SL_i} IG_{\text{ i,s,t}} + \beta^2_{SL_i} t + \beta^3_{SL_i} s,\ \sigma_{SL})$$
$$LT_{i,s,t} \sim \text{T} - \text{Student}(\beta^0_{LT_i} + \beta^1_{LT_i} IG_{\text{ i,s,t}} + \beta^2_{LT_i} t + \beta^3_{SL_i} s,\ \sigma_{LT}, \text{v} = 15)$$

Where an additional coefficient β^3_* is added to control for changes across sequences. When running the full model, we still find an effect of down-weighting (mean = 0.052, SD = 0.019, 94%HDI = [0.019, 0.087]). Moreover, this full model has better model fit than a model that assumes no meta-learning, both in terms of WAIC (11,142 vs. 11,156) and LOO (11,141 vs. 11,156).

Second, some of our dependent variables were correlated. Specifically, looking time is negatively correlated with saccadic latency and look-away probability. Two reviewers observed that this might add erroneous precision to the model. To make sure that the effect of meta-learning was observed for this reason, we run a reduced model that included saccadic latency and look-away, but not looking time. We could still observe the down-weighting effect (mean = 0.037, SD = 0.015, 94%HDI = [0.009, 0.065]), confirming that the correlation between measures was not driving the effect.

Third, data missingness is non-random in our model, as data is missing especially at the end of the sequences and at the end of the task. To make sure that the effects we observe are not solely due to data missing non-randomly, we run an additional model in which we only included the data from the first 10 sequences. Results remained the same, with an effect of meta-learning showing up in terms of down-weighting (mean = 0.064, SD = 0.019, 94%HDI = [0.031, 0.100]). This suggests that the data of the last sequences, which are missing in greater number, and potentially noisier, are not driving the effect we observe.

References

Byers-Heinlein, K., Bergmann, C., & Savalei, V. (2022). Six solutions for more reliable infant research. *Infant and Child Development*, 31(5), e2296.

Gelman, A. (2006). Prior distributions for variance parameters in hierarchical models (comment on article by Browne and Draper). *Bayesian Analysis*, 1(3), 515–534.

Ibrahim, J. G., Chen, M. H., Sinha, D. (2001). *Bayesian survival analysis (Vol. 2)*. New York: Springer.

Rodríguez, G. (2008). Multilevel Generalized Linear Models. In J. de Leeuw & E. Meijer (Eds.), *Handbook of Multilevel Analysis* (pp. 335–376). Springer New York.
